# The infectivity and pathogenicity of a foot-and-mouth disease virus persistent infection strain from oesophageal-pharyngeal fluid of a Chinese cattle in 2010

**DOI:** 10.1186/1743-422X-8-536

**Published:** 2011-12-14

**Authors:** Hui-Fang Bao, Dong Li, Pu Sun, Qiang Zhou, Jun Hu, Xing-Wen Bai, Yuan-Fang Fu, Zeng-Jun Lu, Zai-Xin Liu

**Affiliations:** 1 State Key Laboratory of Veterinary Etiologic Biology, Key laboratory of Animal Virology of Ministry of Agriculture, National Foot-and-Mouth Disease Reference Laboratory of China, Lanzhou Veterinary Research Institute, Chinese Academy of Agricultural Sciences, Lanzhou, Gansu 730046, China; 2 Xinjiang Animal health supervision Institute, Urumuqi 830060, China

**Keywords:** Foot-and-mouth disease virus (FMDV), Persistent infection strain, Oesophageal-pharyngeal fluid, Infectivity, Pathogenicity

## Abstract

**Background:**

Foot-and mouth disease (FMD) is an acute, febrile, and contagious vesicular disease affecting cloven-hoofed animals. Some animals may become persistent infected carriers when they contact FMD virus (FMDV), and persistent infected animals are a dangerous factor to cause FMD outbreak.

**Findings:**

300 OP (oesophageal-pharyngeal) fluid samples were collected from cattle without clinic symptom after one month FMD circulated in 2010 in China. A FMDV strain was isolated when a positive OP sample was passed in BHK21 cell line. The strain, named O/CHN/2010/33-OP, was detected to be O/Myanmar/1998 lineage with VP1 DNA sequence comparison. In order to testify its infectivity, two cattle were challenged with OP fluid and three pigs were put into the same pen for direct contact infection. The result showed that one of the cattle and one of the pigs appeared FMD clinic symptoms respectively. Furthermore, two cattle (three pigs were also put into the same pen for direct contact infection) and three pigs were inoculated with O/CHN/2010/33-OP cell passaged strain. The result showed that one of the challenged pigs appeared FMD clinic symptoms. Two cattle and three pigs in the same pen did not appeared FMD clinic symptoms, but the sera antibody and their OP fluid of two cattle were positive. Meanwhile, the spinal cords of three pigs in the same pen with two cattle were positive detected with multiplex- RT-PCR.

**Conclusion:**

The persistent infection strain O/CHN/2010/33-OP has infectivity and pathogenicity to cattle and pigs, and infected cattle may transmit the virus to pigs although its virulence was lower than the circulated strain O/CHN/Mya98/2010.

## Findings

Foot-and mouth disease is an acute, febrile, and contagious vesicular disease affecting cloven-hoofed animals. Some animals may become persistent infected carriers when they contact FMDV. Van Bekkum detected FMDV aperiodically from oesophageal-pharyngeal sites of cattle without FMD clinic symptoms and gave a concept "persistent infection" [1]. Dave bred some healthy cattle with FMDV-carried ones for several months and the healthy cattle were infected by the same FMDV strain [2]. Another example is that the origin of FMD circulation in Chinese Taiwan in 1999 was FMDV persistent infection carrier of cattle [3]. Therefore, persistent infected animals are a dangerous factor to cause FMD outbreak.

**Figure 1:**
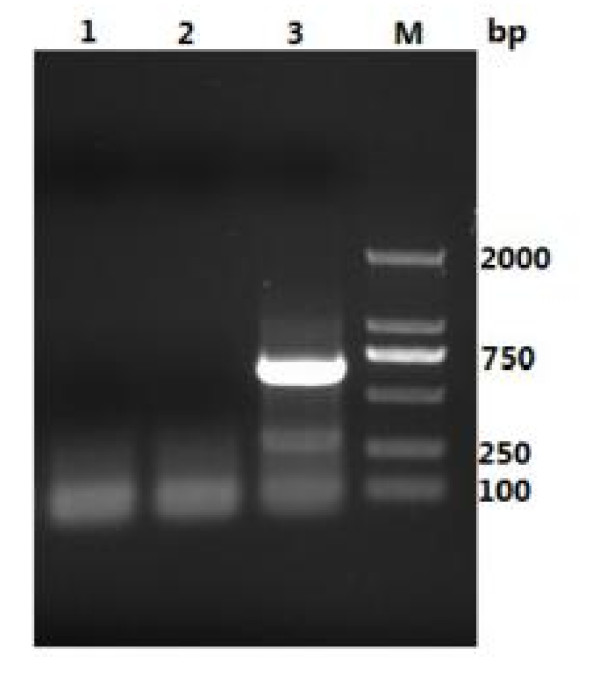
**The test result by multiplex-RT-PCR**. Line 1 and line 2 were two negative samples; line 3 was positive sample; M was DNA marker ladder.

In recent years, Some Asian countries such as China, Mongolia, Korea, Japan populated FMD which were all caused by the O/Myanmar/1998 lineage strain [4,5]. Three hundred OP (oesophageal-pharyngeal) fluid samples were collected from cattle without clinic symptom after one month FMD circulated in 2010 in Xinjiang province of China. A persistent infection strain O/CHN/2010/33-OP was isolated from an OP fluid sample (Figure 1). In this report, we evaluated the infectivity and pathogenicity to cattle and pigs of the strain, and compared the virulence between O/CHN/2010/33-OP and a circulated strain O/CHN/Mya98/2010.

The positive FMDV OP fluid tested with multiplex-RT-PCR was propagated in baby hamster kidney (BHK21) cells serially for seven passages, and the supernatants of infected cells were filtered and stored at -70°C, named O/CHN/2010/33-OP cell strain(1.3 × 10^7.0^PFU/mL). Another cattle tongue FMDV sample was treated with the same way to propagate in BHK21 cells and stored at-70°C, named O/CHN/Mya98/2010 cell strain (1.0 × 10^7.0^PFU/mL).

Total RNA was extracted from samples, using RNeasy Mini kits (QIAGEN) following the manufacturer's instructions. RNA was reverse transcribed into cDNA and amplified using one step RT-PCR kit(TaKaRa). Two primers 1D2(5'-GCG CTG GCA AAG ACT TTG A- 3') and 1D5(5'-GAC ATG TCC TCC TGC ATC TGG TTG A- 3') were needed for VP1 gene's amplification. The PCR products were purified and sequenced (Sunny Biologic Company, Shanghai, China).

**Figure 2:**
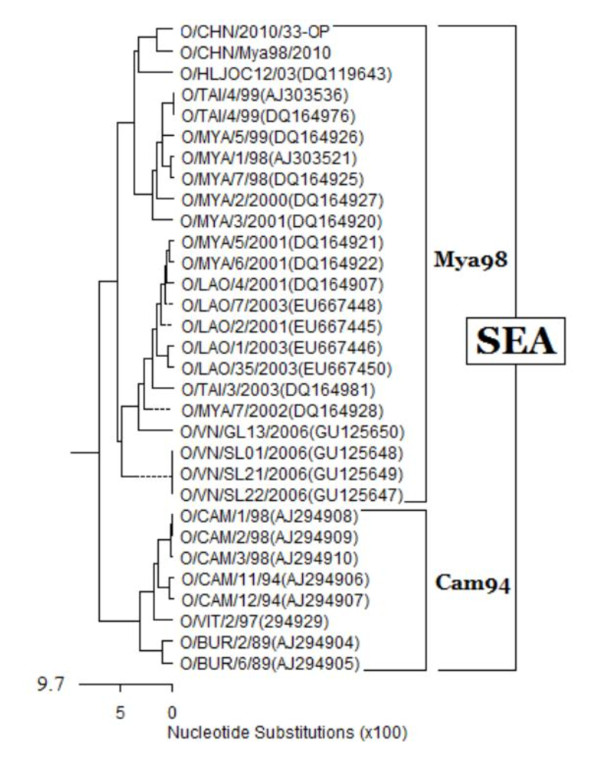
**Multiple sequence alignments using the MegAlign project of DNAStar software package**.

FMDV type O VP1 gene reference sequences were obtained from Genbank on the National Center for Biotechnology Information (NCBI) website (http://www.ncbi.nlm.nih.gov). Multiple sequence alignments were analyzed and percent identities were showed (Figure 2) using the MegAlign project of DNAStar software package (http://www.dnastar.com). The strains, named O/CHN/2010/33-OP and O/CHN/Mya98/2010, were detected to be O/Myanmar/1998 lineage.

Five groups of animals (**Official experimental license number:SYXK[Gansu, China]2010-0003**)were used (**twelve 2-month-old healthy pigs and six 1-year-old healthy cattle, sero-negative for FMDV**) in five separate pens to test the infectivity and pathogenicity of O/CHN/2010/33-OP and O/CHN/Mya98/2010. All infected animals were observed daily for 14 days post inoculation (d.p.i.) before they were slaughtered to collect OP fluids and lymph nodes and spinal cords for virus detection with multiplex- RT-PCR. The first day was recorded at 24 h p.i. (Table 1).

**Table 1:** The infectivity and pathogenicity of O/CHN/2010/33-OP

Group	Infected strain	Animals	dpi	Clinic symptom	Virus in OP fluid	Virus in lymph nodes	Virus in spinal cords	3ABC NSP Ab	Type O SP Ab (LPB-ELISA)
Group 1	O/CHN/ 2010/ 33-OP OP fluid	cattle No.134	8	+				+	1:720
			
			14	+	+	+		+	1:1024
		
		cattle No.184	8	-	-			+	1:360
			
			14	-	-	+		+	1:1024
	
	Direct contact infection	pig No.036	8	-				-	< 1:4
			
			11	+				-	1:90
			
			14	+			+	+	1:512
		
		pig No.039	8	-				-	< 1:4
			
			11	-				-	1:45
			
			14	-			+	-	1:180
		
		pig No.040	8	-				-	1:6
			
			11	-				-	1:45
			
			14	-			+	-	1:128

Group 2	O/CHN/2010/ 33-OP cell strain	cattle No.3	8	-				+	1:90
			
			11	-				+	1: 90
			
			14	-	+	+		+	1: 180
		
		cattle No.146	8	-				-	1:32
			
			11	-				-	1:90
			
			14	-	+	+		+	1:128
	
	Direct contact infection	**pig** **No.041**	**8**	**-**				**-**	**1:4**
			
			**11**	**-**				**-**	**1:4**
			
			**14**	**-**			**+**	**-**	**1:16**
		
		pig No.046	8	-				-	1:12
			
			11	-				-	1:6
			
			14	-			+	-	1:6

		pig No.049	8	-				-	1:4
			
			11	-				-	1:6
			
			14	-			+	-	1:6
		
Group 3	O/CHN/2010/ 33-OP cell strain	pig No.029	4	+				/	/
			
			5	+				/	/
			
			14	+			+	+	1:180
		
		pig No.032	4	-				/	/
			
			5	-				/	/
			
			14	-			+	+	1:512
		
		pig No.033	4	-				/	/
			
			5	-				/	/
			
			14	-			+	+	1:128

Group 4 (control)	O/CHN/Mya98/ 2010 cell strain	cattle No.11	5	-				/	/
			
			8	-				-	1:32
			
			14	+		+		+	1:256
		
		cattle No.12	5	+				-	1:90
			
			8	+				+	/
			
			14	+		+		+	1:180

Group 5 (control)	O/CHN/Mya98/ 2010 cell strain	pig No.047	5	-				/	/
			
			8	-				/	/
			
			14	-			+	+	1:180
		
		pig No.048	5	-				/	/
			
			8	+				-	1:45
			
			14	+			+	+	1:360
		
		pig No.050	5	+				/	/
			
			8	+				-	1:180
			
			14	+			+	+	1:360

Note: / undetected + positive - negative

dpi : days post infection NSP: nonstructural protein SP: structural protein

Ab: Antibody

In the first group, two cattle (No.134, 184) were injected 0.6 ml positive OP fluid in the surface of their tongue respectively, and three pigs(No.036,039,040) were bred in the same pen. One of the cattle (No.134) developed blister in the foot on the day 8 p.i.. The sera titer of both cattle was 1:1024 on the day 14 p.i. although No.184 did not show clinic symptom. Pig No.036 developed blister on the day 11 p.i.. The sera titer of three pigs were 1:512,1:180, 1:128 respectively on the day 14 p.i.. They were all positive.

In the second group, two cattle (No.3, 146) were injected 2 ml O/CHN/2010/33-OP cell strain in the surface of their tongue respectively, and three pigs (No.041, 046, 049) were bred in the same pen. The OP fluids of both cattle were positive on the day 14 p.i. although clinic symptoms did not appear. The sera titer of both cattle was 1:180 and 1:128 on the day 14 p.i.. Lymph nodes and spinal cords of three pigs were FMDV positive although clinic symptoms did not appear on the day 14 p.i..

In the third group, three pigs (No.029, 032, 033) were injected 2 ml O/CHN/2010/33-OP cell strain into one heel bulb and into the ear-root-neck area respectively. Pig No.029 developed primarily blister in the injected foot on the day 4 p.i. and developed secondary blister in another foot on the day 5 p.i.. Its sera titer was 1:180. The sera titer of pigs No.032 and No.033 was 1:512, 1:128 respectively although clinic symptoms did not appear.

In the fourth group, two cattle (No.11, 12) were injected 2 ml O/CHN/Mya98/2010 cell strain in the surface of their tongue respectively. Both cattle developed blister.

In the fifth group, three pigs (No.047, 048, 050) were injected 2 ml O/CHN/Mya98/2010 cell strain into one heel bulb and into the ear-root-neck area respectively. Only pig No.047 did not appear clinic symptom. Other two pigs developed blister on the day 5~7 p.i..

From above experimental result, the persistent infection strain O/CHN/2010/33-OP may infect other cattle and pigs. That FMDVs were found out from spinal cords of five direct contact infected pigs (group 1 and group 2) means the virus transmitted from cattle to pigs, and one pig appeared blister means the virus has pathogenicity and can lead to illness of pig. One cattle appeared blister in group 1 when injected OP fluid containing virus. The possible reason was that individual difference may induce some cattle's clinic symptom but some not. This suggested that the persistent infection strain O/CHN/2010/33-OP can infect the same type of animals.

The pathogenicity of the persistent infection strain O/CHN/2010/33-OP was weaker than the popular strain O/CHN/Mya98/2010. Two cattle (group 2) inoculated with the O/CHN/2010/33-OP cell strain did not appear clinic symptom but other two ones (group 4) infected with the popular strain O/CHN/Mya98/2010 developed blister. Meanwhile, only one of three pigs (group 3) infected with the O/CHN/2010/33-OP cell strain appeared clinic symptom but two of three pigs (group 5) infected with the popular strain O/CHN/Mya98/2010 appeared clinic symptom. The dose and quantity of the inoculated virus was the same. These data demonstrated that the virulence of the persistent infection strain O/CHN/2010/33-OP was lower than the popular strain O/CHN/Mya98/2010, which was similar with a previous report about another FMDV persistent infection strain [6].

In brief, the persistent infection strain O/CHN/2010/33-OP has infectivity and pathogenicity to cattle and pigs, so it is very important to detect FMD persistent infected animals and eliminate carriers in order to control outbreak of FMD.

## Competing interests

The authors declare that they have no competing interests.

## Authors' contributions

ZXL was responsible for the research. HFB, DL and PS carried out most of the experiments. DL wrote and revised the manuscript. JH, QZ, XWB, YFF, ZJL carried out part of the experiment. All of the authors read and approved the final version of the manuscript.
